# From Words to Wounds: Cyberbullying and Its Influence on Mental Health Across the Lifespan

**DOI:** 10.3390/bs15050619

**Published:** 2025-05-02

**Authors:** Sofia von Humboldt, Gail Low, Isabel Leal

**Affiliations:** 1 William James Center for Research, Instituto Universitário de Ciências Psicológicas, Sociais e da Vida—Instituto Universitário, 1149-041 Lisbon, Portugal; ileal@ispa.pt; 2 Faculty of Nursing, International Health Research, MacEwan University, Edmonton, AB T5J 4S2, Canada; lowg4@macewan.ca

**Keywords:** cyberbullying, lifespan, mental health, qualitative study

## Abstract

Cyberbullying can be prevalent across different life stages, with lasting traces on mental health across the lifespan. This study aims to (a) explore how cyberbullying is emotionally experienced across three distinct age groups and (b) analyze the influence of cyberbullying on mental health across the lifespan. This study included 883 participants divided into three age groups: 18–39, 40–59, and 60+. In-depth semi-structured interviews were conducted to gather participants’ experiences and perspectives. The data were then subjected to content analysis, which revealed a number of themes. The first objective revealed the following themes: For ages 18–39: (a) feeling ashamed or humiliated (92.4%), (b) withdrawing from friends and family, and (c) experiencing harassment as positive and difficulties with rules. For ages 40–59: (a) losing interest in hobbies (89.5%), (b) questioning about things they did or did not do, and (c) experiencing a sense of missing out. For ages 60+: (a) negative thoughts and self-talk (91.3%), (b) feeling judged negatively, and (c) feeling financially vulnerable. The second objective showed: For 18–39: (a) depressive symptoms (79.7%), (b) easy anger, and (c) suicidal behavior. For 40–59: (a) anxiety (93.2%), (b) low self-esteem, and (c) the use of substances. For 60+: (a) frustration (78.1%), (b) isolation, and (c) disturbances in sleep and eating patterns. This study highlights the significant psychological and emotional impact of cyberbullying across age groups, emphasizing the need for targeted interventions that address the unique challenges faced by individuals at different life stages. The findings underscore the importance of developing age-specific strategies to mitigate the effects of cyberbullying and to have perpetrators take responsibility for their reckless disregard for others, and ultimately, themselves.

## 1. Introduction

### 1.1. The Concept of Cyberbullying

Cyberbullying has been extensively studied in recent years and encompasses individuals or groups repeatedly sending hostile or harmful messages to cause discomfort or harm to others (Tokunaga, 2010). Olthof et al. (2011) frame cyberbullying as a calculated behavior designed to dominate others. Technology has afforded new avenues for scams, threats, and harassment (NORC, 2021), creating a shift in how individuals experience and respond to such challenges across the lifespan. This can manifest in various forms, such as mean messages sent via mobile phones, email, or social media; spreading malicious rumors, images, or videos online; and sexting, where sexually suggestive content is circulated to humiliate someone (Balakrishnan, 2015). Cyberbullying, a digital extension of traditional bullying, is characterized by an imbalance of power, repeated harmful behavior, and intent to victimize (Marinoni et al., 2024a). The terminology for technology-facilitated abuse includes cyberbullying, online bullying, cyber abuse, and cyberstalking. Common to these behaviors is the use of technology—such as emails, text messages, videos, images or social media—to harass, threaten, intimidate, alienate, humiliate, extort, or defraud others (NORC, 2021).

Some authors, such as Waasdorp and Bradshaw (2015) and Wolke et al. (2017), argue that cyberbullying shares key traits with traditional bullying, including intentional harm, repeated aggression, and an imbalance of power. Others emphasize its unique characteristics. For example, unlike face-to-face bullying, cyberbullying often remains visible indefinitely, is not confined to a specific location, and can be carried out anonymously, making it difficult for victims to escape its effects (Kowalski et al., 2014). The anonymity of online interactions allows perpetrators to act without immediate consequences, gaining attention while shielding their identity. Additionally, engaging in cyberbullying requires a certain level of digital literacy, as it relies on the ability to navigate online platforms and communication technologies. Since online interactions lack the direct, real-time social cues present in traditional bullying, the motivations and behaviors behind cyberbullying often follow different patterns (Marinoni et al., 2024b).

### 1.2. Cyberbullying Across the Lifespan

Cyberbullying is particularly prevalent among youth, with many experiencing both cyberbullying and traditional bullying simultaneously, intensifying the risks involved (Waasdorp & Bradshaw, 2015). Research by Li et al. (2024) indicates that cyberbullying may signify broader social difficulties, as most victims also experience traditional bullying. Moreover, younger generations, having grown up in a digital-first world, are not only more frequent internet users but also engage more deeply with social media, increasing their vulnerability to cyberbullying (Schimmele et al., 2021; Wavrock et al., 2021). For young adults aged 18 to 29, cybervictimization rates are significant, with behaviors such as receiving unwanted explicit content (15%) and aggressive messages (13%) being the most common. Women, particularly, face higher rates of online abuse, including appearance-based harassment focusing on body shape and size (Hango, 2023; Prince et al., 2024).

Among older adults, cyber abuse is also a growing concern, with 63% of individuals over 50 in the United States reporting at least one form of cyber abuse, often in the form of financial threats (NORC, 2021). However, younger adults remain more susceptible to cyberbullying, with prevalence peaking in adolescence and early adulthood before tapering in the late 20s (Hango, 2023; Wang et al., 2019). Increased internet usage, particularly prolonged daily exposure or activities like social networking and gaming, strongly correlates with higher rates of cyberbullying and victimization (Balakrishnan, 2015; Hango, 2023). Victims often respond by altering their online and offline behaviors, such as blocking perpetrators, restricting internet use, or carrying protective items. Additionally, many victims of cyberbullying report overlapping experiences of other forms of victimization, including stalking and physical or sexual assault. These are very real dangers (Hango, 2023).

A cyclical relationship between victims and perpetrators is evident, as cybervictims often become cyberbullies themselves (Balakrishnan, 2015). Social connections may serve as a protective factor; individuals with more close friends may be less likely to be targeted or may better cope with the aftermath of victimization. Conversely, those with fewer social ties may be more vulnerable to cyberbullying, as they are perceived as easier targets (Kendrick et al., 2012). Additionally, cyberbullying can also involve controlling others through criticism, manipulation, or covertly demeaning behaviors. Individuals may engage in controlling behaviors online, offering unsolicited opinions or jokes that mask envy or a desire for dominance. Perhaps an inferior self is at play (Favini et al., 2023; von Humboldt et al., 2022b).

### 1.3. Cyberbullying Impact on Mental Health

Although social media enables rapid access to information and seamless communication, it is also associated with cyberbullying, unsafe online activities, social isolation, psychological distress, and aggressive behavior (Kennedy et al., 2025). Due to advancements in social media platforms, instant messaging, and widespread internet accessibility, traditional bullying has expanded, adding a virtual dimension to face-to-face bullying (Kennedy et al., 2025). The internet has become a popular platform for social interaction, offering anonymity that enables harmful behaviors (Balakrishnan, 2015). Anonymity enables a lack of ownership and invites exacerbated disregard. Bullying, whether in person or online, is a significant risk factor for both physical and mental health (Li et al., 2024). Cyberbullying in particular can have severe repercussions. Victims of cyberbullying often experience anxiety, stress, depression, low self-esteem, and substance use (Agustiningsih et al., 2024). In extreme cases, it can lead to isolation and suicidal behaviors, including suicidal thoughts, attempts, and completion. Anonymity exacerbates mental harm and steals lives (Agustiningsih et al., 2024; Balakrishnan, 2015; Li et al., 2024).

One distinguishing feature of cyberbullying is the anonymity of the perpetrator, which can exacerbate the situation. The public nature of online bullying, coupled with the reduced sense of guilt and ethical responsibility on the Internet, means that the consequences can be far-reaching. Furthermore, the permanence and spread of online content make it difficult for victims to control or predict the duration and impact of the bullying and the harm it does can last indefinitely due to the internet’s perpetual memory (Kowalski et al., 2014). In contrast, traditional bullying typically involves identifiable perpetrators, and the bullying itself is often easier to anticipate and avoid, contributing to the more enduring and damaging effects of cyberbullying (Li et al., 2024).

The impact of cyberbullying on young people’s mental health is profound, leading to increased social anxiety and withdrawal (Coelho & Romão, 2018), body dissatisfaction and eating disorders (Day et al., 2022; Ramos Salazar, 2021), generalized anxiety and depression (Maurya et al., 2022), sleep problems (Nagata et al., 2023), obsessive-compulsive problems (Moretta & Buodo, 2021), and a decrease in overall psychological well-being (Foody et al., 2019). Cyberbullying victims exhibit heightened levels of anxiety and depression (Hemphill et al., 2015), and are at a greater risk of self-harm and suicidal behavior compared to those who experience traditional bullying (Heerde & Hemphill, 2019). E-bullies must take ownership of and be held accountable for their mental and emotional toll on others.

Adults often experience cyberbullying in more complex contexts, such as within professional or social circles. Work-related online bullying can be particularly damaging, as it can undermine job satisfaction, erode confidence, and increase workplace stress (Kowalski et al., 2018). For those who experience it in professional settings, the consequences can extend beyond personal distress, potentially threatening career advancement and professional relationships (Wang et al., 2019).

In relation to older adults, Tripathi et al. (2019) explored the various forms of cyber-victimization, particularly its financial and emotional toll. Older victims of online fraud often suffer significant losses, including retirement savings and funds reserved for health-related emergencies. The absence of support from authorities such as the police and financial institutions only deepens the emotional distress, leading to feelings of shame, anxiety, and depression (Tripathi et al., 2019; von Humboldt et al., 2023c, 2023d). Older individuals who experience cyber-victimization may also encounter social isolation, marginalization, and stigma. LGBTQ+ older adults are often subjected to additional forms of harassment, including discrimination and offensive remarks, which further exacerbate the emotional impact of the experience (Tinney et al., 2015; von Humboldt et al., 2021, 2022b).

### 1.4. Current Study

While previous research on cyberbullying has predominantly focused on its prevalence among teenagers and young adults (Wang et al., 2019), little is known about how it manifests and affects older individuals. Recent studies have also predominantly centered on single age groups or specific forms of online harm, creating a gap in understanding the broader, cross-age impact of cyberbullying (Agustiningsih et al., 2024; Day et al., 2022; Favini et al., 2023; Prince et al., 2024). Furthermore, no studies have compared its impact across different life stages. By adopting a comparative approach, this study sheds light on the unique and shared challenges faced by individuals in these age groups, offering critical insights into how cyberbullying influences mental health in diverse demographic contexts. This study addresses this critical gap in the existing literature by exploring the experiences of cyberbullying across three distinct age groups (18–39, 40–59, and 60+) and examining its effect on mental health. Therefore, this study’s objectives were to (a) explore how cyberbullying is emotionally experienced across three distinct age groups and (b) understand the influence of cyberbullying on mental health across the lifespan. Targets of cyberbullying had louder-than-perpetrator voices, and rightly so (United Nations, n.d.).

## 2. Method

### 2.1. Recruitment and Sampling

Participants were selected based on specific eligibility criteria: (1) aged 18 or older, (2) capable of making an informed decision, (3) no history of cognitive impairment, psychiatric or neurological disorders, or substance abuse, and (4) familiarity with modern technologies like smartphones, tablets, computers, and apps. Recruitment utilized a combination of snowball and convenience sampling, which helped target both easily accessible individuals and those referred by initial participants (Sarker & AL-Muaalemi, 2022). The study aimed for diversity by recruiting through various channels, such as senior universities, community centers, social media, mailing lists, and public message boards in metropolitan areas in Portugal. Communication methods included phone calls, email, and in-person interactions, tailored to participants’ preferences. A total of 950 individuals provided informed consent, and 883 participants (aged 18–91 years, *M* = 46.4; *SD* = 6.841) were included. Participants were categorized into three age groups: 18–39 years (34.1%), 40–59 years (33.3%), and 60+ years (32.6%). The diversity among age groups was achieved by purposively selecting participants from each age group while considering the key demographic factors like gender, education, marital status, living arrangements, family annual income, and perceived health. Table 1 presents the socio-demographic profile of the sample, showcasing diversity in age, education, and marital status.

Semi-structured interviews, lasting around 35 min, were conducted between 1 June and 30 September 2024, until data saturation was reached. Power analysis applied to classical content analysis was ensured through theoretical saturation, which certifies that redundant elements were systematically (correctly) eliminated, leaving only mutually exclusive themes. This approach prevented thematic overlap while ensuring no conceptual gaps remained. Content analysis was applied to identify and refine these themes, aligning with established methodological frameworks (Wutich et al., 2024; Hennink & Kaiser, 2022). This process ensured that the final themes captured distinct and meaningful aspects of the data, without repetition. These interviews combined predefined questions with the flexibility to explore emerging topics based on participants’ responses. This approach allowed for a structured, yet adaptable conversation, ensuring that key themes are covered while also capturing participants’ unique perspectives. The interview guide, developed by the authors, included two questions: “Could you describe your emotional experiences about cyberbullying?”; and “In your opinion, how does cyberbullying influence your mental health?”. Interviews were conducted via platforms like Skype, SurveyMonkey, Zoom, and WhatsApp, ensuring accessibility and flexibility. Audio recordings were made to accurately capture the data, and participants were assured of their anonymity. Digital communication confidentiality was safeguarded by utilizing secure platforms that offer end-to-end encryption to protect participants’ privacy. Additionally, all sessions were conducted privately, and access was restricted to authorized individuals only. Participants were informed about the confidentiality measures in place and provided their consent prior to being interviewed. A safe interview setting was paramount, for our ethical approval granted by the Ethics Committee of William James Center for Research and ISPA -Instituto Universitário (SFRH/BPD/116114/2016 11 July 2016). Our study also adhered to the principles of the 1964 Helsinki Declaration and its amendments.

### 2.2. Data Analysis

We employed content analysis, following the framework by Erlingsson and Brysiewicz (2017), to systematically analyze in-depth interviews conducted via telephone and online platforms. The study adhered to the COREQ checklist (Tong et al., 2007) to ensure transparency and methodological rigor.

Content analysis (Erlingsson & Brysiewicz, 2017) was used to analyze the qualitative data, employing a deductive framework aligned with the research questions. A codebook was developed to categorize the data, which was then organized into meaningful themes using category matrices. The categorization process focused on ensuring distinct, relevant, and cohesive themes. Three psychologists independently reviewed and approved the classification, ensuring the principles of homogeneity, relevance, and objectivity were upheld. All participants’ voices were deservedly represented (United Nations, n.d.).

**Figure 1:**
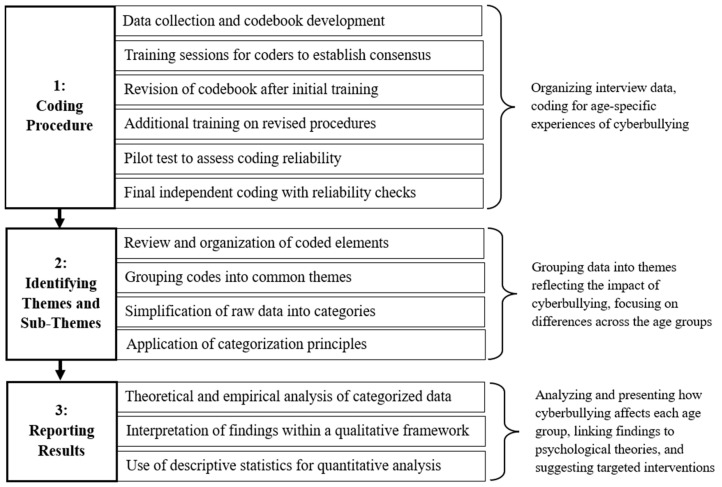
Comprehensive process for content analysis.

Frequency counts were used to determine the prevalence of specific themes within the data. To ensure the reliability and validity of the coding process, three researchers independently coded the interviews. Inter-rater reliability was calculated using Cohen’s kappa coefficient, with a score of *k* = 0.91, indicating substantial agreement. The process of maintaining inter-rater reliability involved regular discussions and recalibration among the coders to resolve discrepancies and ensure consistent interpretation of the themes. The qualitative analysis incorporated both descriptive and theoretical frameworks. A two-coder system was employed to maintain methodological rigor (Neuendorf, 2002). Descriptive quantitative analysis of sociodemographic data (means, medians, percentages, and frequencies) was performed before conducting the qualitative analysis. Statistical analyses were considered significant at *p* ≤ 0.05. The dual-coding process, illustrated in Figure 1, reinforced the robustness of the study’s findings.

## 3. Results

The first objective of this study was to explore how cyberbullying was experienced by individuals across three age groups (see Table 2). For the 18–39 age group, the identified themes included the following: (a) feeling ashamed or humiliated (92.4%), (b) withdrawing from friends and family (81.1%), and (c) experiencing harassment as positive and difficulties with rules (80.7%).

### 3.1. Theme 1 [Age 18–39]: Feeling Ashamed or Humiliated

Participants most frequently (92.4%) reported feeling ashamed or ridiculous. This theme reflects the emotional burden participants experienced due to cyberbullying, where they often felt humiliated, degraded, or belittled. Moreover, they subsequently self-identified with lower self-esteem and self-worth.

Maria shared, “I love posting videos of myself singing; it’s something that truly brings me joy. But once, someone created a montage with my face and a pig, and soon everyone started calling me ‘Miss Piggy’. People mocked my appearance and ridiculed my voice. It felt like no matter what I did, no matter how much I tried to be myself, they would find something to criticize. It feels like even if I breathe, they’ll have something wrong to say about me” (Maria, female, 24 years old). This emphasizes the constant scrutiny and harsh judgment that comes with cyberbullying, which profoundly impacts the victim’s sense of self and expression.

John recounted, “There was once an anonymous person who called me ‘a disgrace to humanity’ for sharing my thoughts on a topic I cared about. That one phrase haunted me for weeks. Every time I tried to talk about something, I’d hear that voice in my head telling me I was a disgrace, telling me to just shut up. Someone I don’t even know, and who definitely doesn’t know me, made me feel like I wasn’t even human anymore, like my very existence was a joke” (John, male, 31 years old). This illustrates how cyberbullying can leave lasting emotional wounds and cause the victim to internalize such hurtful comments, deeply affecting their sense of identity.

### 3.2. Theme 2 [Age 18–39]: Withdrawing from Friends and Family

The second most identified theme among participants was the tendency to withdraw from friends and family (81.1%). This theme highlights how cyberbullying led to feelings of isolation and avoidance of close relationships, as participants often felt ashamed, hurt, or unworthy of social interaction. These individuals chose to distance themselves from loved ones, fearing judgment, rejection, or feeling that others would not understand the emotional toll of their experiences.

Carlos shared the emotional strain felt, pushing the individual to isolate himself from supportive social networks: “I couldn’t face my friends anymore. I was sure that they had seen the same comments I did online, and I started to wonder if they thought the same way as those people bullying me. I couldn’t shake the feeling that they might be judging me too, that maybe they were just pretending to be supportive. It made me feel like I couldn’t trust anyone anymore” (Carlos, male, 29 years old).

Another participant, Maria, expressed, “I couldn’t talk to my family about it, not even my sister. They wouldn’t understand. I started spending more time alone in my room. My parents told me to log off, but they don’t understand that if I’m not online, I don’t exist for others. It was easier to just hide away from everyone” (Maria, female, 18 years old). Withdrawal from loved ones can be a means to avoid others’ judgment and further emotional harm.

### 3.3. Theme 3 [Age 18–39]: Experiencing Harassment as Positive and Difficulties with Rules

Experiencing harassment as positive and difficulties with rules emerged as a significant experience for 80.7% of participants. This theme reflects how cyberbullying sometimes influenced individuals to develop a skewed perception of aggression and authority, taking it as if there were no consequences. The constant harassment led some to rationalization or normalization of such behavior, while others became disillusioned with societal rules as they struggled with the emotional toll of bullying.

Lucas explained, “After all the insults and harsh comments online, I started to think maybe the only way to get respect was to fight back—not just with words, but by posting more aggressive things myself. I’d respond to people’s comments with threats, calling them out and attacking their appearance. I thought if I didn’t do that, I would be seen as weak. No one stepped in to stop the bullying either, so I started to feel like fighting fire with fire was the only way to survive” (Lucas, male, 38 years old). This illustrates how Lucas began to adopt a more aggressive online persona, using the same methods that had been used against him as a form of defense, as he lost trust in the system to intervene.

Another participant, Ana shared, “I’ve always followed the rules, but after experiencing so much bullying online, it felt like no one cared about me. I’d report the comments, but nothing ever changed. It started to feel like the rules didn’t matter—why should I follow them if no one else was? I began posting things that would normally get flagged, because it felt like if I didn’t take control, I’d be treated like a doormat” (Ana, female, 28 years old). Her frustration and sense of abandonment led her to question not just her self-worth but the purpose of following rules in a space where her pain seemed to be ignored.

In the 40–59 age group, the themes encompassed: (a) losing interest in hobbies (89.5%); (b) questioning about things they did or did not do (76.9%); and (c) experiencing a sense of missing out (74.8%).

### 3.4. Theme 1 [Age 40–59]: Losing Interest in Hobbies

The most frequently mentioned theme (89.5%) was losing interest in the things you love. This theme reflects how cyberbullying led to a gradual disengagement from activities that once brought joy or fulfillment. Many participants described how the emotional toll of these experiences dampened their motivation and enthusiasm.

One participant, Clara, shared, “I used to love painting and sharing my work online. But after people started leaving cruel comments, calling my art ‘childish’ or saying it looked like something a five-year-old would make, I tried changing my style to avoid more criticism. The problem is it didn’t feel like my art anymore. What used to make me happy became something I don’t feel like doing. Now, I avoid it altogether” (Clara, female, 53 years old). Clara’s quote illustrates the internalized impact of bullying. External criticism led to a loss of confidence and passion for her creative outlet.

Another participant, Alex, recounted, “Music was everything to me. I loved playing guitar and even posted covers on social media. But after a group of people started saying I should ‘save everyone’s ears’ and that I sounded like a dying animal, I just couldn’t do it anymore. My guitar’s still in the corner of my room, but I can’t bring myself to touch it. They ruined it for me” (Alex, male, 49 years old). This experience highlights how cruel online remarks can strip individuals of their connection to beloved hobbies, leaving them with lingering feelings of inadequacy and shame. Isn’t leisure meant to enhance (versus harm) our mental health?

### 3.5. Theme 2 [Age 40–59]: Feeling Guilty About Things They Did or Did Not Do

A good proportion (76.9%) of midlife adults felt guilty about things they did or did not do. They experienced emotional negativity and blamed themselves either for the actions that they took to circumvent or their inability to respond to cyberbullying. Many described carrying a deep sense of regret that was amplified by the public nature of online interactions.

One participant, Helena, reflected, “A colleague filmed me in the bathroom at work and spread the video around. Later, she came to me saying she felt guilty about what happened. But all the shame is on me. The video is online, it’s permanent, and my reputation is damaged, while hers is untouched. I feel like I’m the only one suffering the consequences” (Helena, female, 57 years old). Her experience highlights how bullying can lead to a vicious cycle of inaction and self-blame, with victims internalizing responsibility for harassment.

Another participant, Liam, recounted, “I once replied to a nasty comment, thinking I could shut them down, but it just made things worse. They twisted my words, making me look like the bad guy. I keep thinking I should have handled it differently, or maybe not said anything at all. It’s like no matter what I do, I’m in the wrong” (Liam, male, 46 years old). His account underscores the complexities of responding to bullying, where attempts to defend oneself can sometimes exacerbate the situation, leaving victims trapped in regret and second-guessing their actions.

### 3.6. Theme 3 [Age 40–59]: Experiencing a Sense of Missing out

Feelings of missing out emerged as the next prevalent theme among 74.8% of participants, illustrating how cyberbullying not only affects self-esteem but also disrupts social connections, leading to feelings of exclusion and detachment from shared experiences. This sense of being left out often stems from withdrawing to avoid further harm, which unintentionally deepens the gap between the individual and their social circle. This sense of missing out was characterized by these individuals’ perception of being excluded from important social interactions. Participants described instances where cyberbullying led them to believe they were being left out of social events or activities, which in turn heightened their emotional distress and further isolated them from their peers.

Philip described this vividly, saying, “I used to love joining family video calls and catch-ups. It was how I stayed close to my kids, especially since they’re all grown up and live far away. But after the bullying started, I stopped joining in. I’d see pictures of family events later—smiling faces, inside jokes—and feel like I didn’t belong anymore. They stopped inviting me, and honestly, I don’t blame them. Who wants someone like me dragging the mood down?” (Philip, male, 61 years old). His reflection captures how bullying fosters isolation, further feeding the perception of exclusion from meaningful family moments.

Similarly, Paula explained, “Every time I’d scroll through social media, I would see my friends posting about events or sharing memories together. And there I was—sitting at home, too scared to be part of it. It felt like life was happening without me. Even when they reached out, I just couldn’t bring myself to join. It’s like the bullying had built an invisible wall, and I was stuck on the outside” (Paula, female, 47 years old). Paula’s account highlights the self-perpetuating nature of exclusion, where fear of further bullying creates a cycle of social detachment and missed connections.

For individuals aged 60 and older, the themes included: (a) negative thoughts and self-talk (91.3%), (b) feeling judged negatively (87.5%), and (c) feeling financially vulnerable (86.1%).

### 3.7. Theme 1 [Age 60+]: Negative Thoughts and Self-Talk

The most verbalized (91.3% of participants) theme was negative thoughts and self-talk. Cyberbullying can deeply impact an individual’s inner dialogue, often leading to destructive thought patterns and a skewed sense of self-worth. Participants described how harassment not only affects their confidence but also reinforces feelings of inadequacy and self-doubt. This constant negative self-reflection is both a direct consequence of the bullying and a persistent reminder of its emotional toll.

Tim wanted to interact with people online, “I thought it would be nice to connect with others, share my thoughts, and feel part of something. But then, I started getting these messages from people who didn’t even know me, calling me ‘irrelevant’ and ‘out of touch.’ It made me question myself, like maybe they were right. After a while, I stopped posting altogether, but the messages still lingered in my mind. It made me feel like I was too old to have a voice anymore” (Tim, male, 67 years old).

Similarly, Sandra expressed, “There was a time I really tried to be positive, but the comments online made me feel like I was trapped in my own head. Every time I looked in the mirror, I saw everything that was wrong with me. ‘You’re too old,’ ‘You don’t matter,’ ‘You’re just a burden.’ Those were the thoughts that kept coming up. It wasn’t just the bullies online—it was me, too, constantly telling myself I wasn’t good enough to even be here” (Sandra, female, 71 years old). Sandra tells us how bullying can trigger self-criticism and feelings of unworthiness and even invisibleness. One can become his own worst enemy.

### 3.8. Theme 2 [Age 60+]: Feeling Judged Negatively

Feeling that you are being judged negatively was the second most (87.5%) verbalized theme. This theme captures the participants’ pervasive sense of being scrutinized and judged harshly in online spaces, particularly when interacting with younger generations or sharing personal opinions. For many, this judgment extended to assumptions about their age, abilities, and relevance, leaving them feeling unwelcome in digital environments.

Lauren shared her experience of joining an online discussion group, hoping to exchange ideas and learn from others: “I posted a comment about my thoughts on a book I loved, and someone immediately replied, ‘Typical old-fashioned thinking. Do you even understand modern literature?’ It wasn’t just the words; it was the tone, like I didn’t belong there. It made me feel like online world is not for me, an older woman” (Lauren, female, 76 years old). This comment illustrates how such remarks discourage older adults from engaging, fostering feelings of alienation.

Lando reflected on his decision to stop sharing photos of his family online: “I used to love posting pictures of family gatherings, but then I saw comments like, ‘Why do older people post so much? No one cares about your outdated life.’ I mean, it’s true—I don’t need to post it, but I wanted to share this nice moment with others. Why can my grandson do it, and I can’t?” (Lando, male, 68 years old). His experience highlights how judgment online can suppress the joy of sharing meaningful moments.

### 3.9. Theme 3 [Age 60+]: Feeling Financially Vulnerable

Feeling financially vulnerable emerged as the next significant concern for 86.1% of participants, reflecting the intersecting nature of cyberbullying and financial insecurity. Many participants described how they were targeted financially by cyberbullies, which exacerbated their feelings of inadequacy and worries about their future monetary stability.

Manuel shared his struggle after receiving mocking comments about his financial limitations: “I once mentioned in a group discussion that I couldn’t afford the latest phone, and someone responded, ‘Of course, you’re retired. Isn’t it time to let the younger ones enjoy life?’ It stuck with me. It’s not just about the phone—it’s the way they make you feel small, like you don’t belong in this world anymore” (Manuel, male, 71 years old). This interaction intensified his anxiety about keeping up with modern technology and societal expectations.

Ellen, another participant, described how she fell victim to a financial scam that exploited her desire to connect with others: “I joined what I thought was a friendly group offering advice on saving money. They seemed so helpful at first, but then someone convinced me to invest in this scheme, saying it would double my savings. They told me, ‘At your age, you need to think about making the most of what you have.’ I trusted them, and I lost almost everything I put in. It wasn’t just the money—it was the humiliation of being tricked, of being seen as naive because I’m older” (Ellen, female, 67 years old). This experience left her distrustful and hesitant to engage in online communities, fearing further exploitation.

The second objective aimed to examine the impact of cyberbullying on mental health across the same age groups (see Table 2). Among participants aged 18–39, the findings highlighted: (a) depressive symptoms (79.7%); (b) easy anger (76.1%); and (c) suicidal behavior (43.2%).

### 3.10. Theme 1 [Age 18–39]: Depressive Symptoms

Depressive symptoms provoked by cyberbullying was the most common (79.7%) theme among the youngest participants. The relentless negativity they faced online seemed to seep into their offline lives, eroding their sense of self-worth and contributing to persistent feelings of sadness and hopelessness.

Joanne, for instance, shared how online criticism affected her self-perception: “I used to be so confident about my work. I’d post my illustrations online, and they’d get good feedback. But then, one day, a string of comments came in, calling my art ‘lazy’ and me ‘delusional for thinking I could ever make it.’ I started doubting everything, not just my art but my abilities in general. It became hard to even get out of bed, let alone create anything” (Joanne, female, 32 years old).

Mike explained how the bullying isolated him emotionally: “They called me a failure, a loser. At first, I laughed it off, but those words stayed with me. I stopped reaching out to my friends because I felt like they probably saw me the same way. Eventually, it was just me, my thoughts, and this overwhelming sadness that I couldn’t shake off” (Mike, male, 28 years old). These accounts underline the profound impact cyberbullying has on young adults’ mental health, where constant exposure to negativity can lead to persistent depressive symptoms, affecting their daily lives and aspirations.

### 3.11. Theme 2 [Age 18–39]: Easy Anger

For 76.1% of participants, the experience of cyberbullying often resulted in heightened irritability and a tendency to respond with anger, both online and in their personal lives. The constant barrage of hurtful comments created an emotional vulnerability, making it harder to regulate their reactions.

Sophia explained how cyberbullying impacted her emotional state: “I used to be the kind of person who would let things slide. But after reading those comments—calling me ugly, stupid, or even worse—I started feeling this anger rising inside me. It felt like I was constantly ready to explode, even over the smallest things. I’d argue with my friends over nothing, and I hated myself for it. It was like their words were eating at me from the inside out” (Sophia, female, 25 years old).

Colin shared a moment where his anger overtook his usual restraint: “I got a comment saying, ‘You’re nothing but a clown, pretending to know anything.’ It got under my skin. I wrote this long, furious reply, attacking the person back. But even after posting it, I wasn’t satisfied. It was like their words had unlocked something ugly in me, and I hated it” (Colin, male, 30 years old). These narratives show how cyberbullying can provoke a cycle of anger, where individuals struggle to process their emotions healthily, leading to strained relationships and regret over impulsive reactions.

### 3.12. Theme 3 [Age 18–39]: Suicidal Behavior

The impact of cyberbullying went beyond emotional distress, with 43.2% reporting suicidal thoughts or behaviors as a consequence of the relentless harassment. The harsh online interactions eroded their sense of self-worth and led them to feel trapped in a cycle of negativity, coupled with the isolation it created, led them to contemplate ending their lives as a way to escape their pain.

Anabella shared her painful experience: “I kept thinking: ‘Maybe the comments were right, maybe I wasn’t worth anything.’ I felt watched, judged, and mocked every time I posted something. I began questioning my purpose, wondering if it would be better if I just disappeared. I thought no one would miss me” (Anabella, female, 30 years old).

Tim described the emotional toll of losing friends: “It started with small things—friends stopped reaching out, stopped talking to me. I felt like I was the problem, like I was just a burden to them. The more they pulled away, the more I felt isolated. One day, I couldn’t take it anymore. I felt like I had no one to turn to. I couldn’t share how I felt, and the only way out seemed to be ending it all. I thought nobody cared about me anymore” (Tim, male, 34 years old). These experiences illustrate the deep emotional impact cyberbullying had on younger individuals, highlighting the sense of isolation, the loss of social support, and the overwhelming thoughts of suicide that emerged because of constant cyberbullying.

For those aged 40–59, the identified effects were: (a) anxiety (93.2%); (b) low self-esteem (76.2%), and (c) the use of substances (74.8%).

### 3.13. Theme 1 [Age 40–59]: Anxiety

Anxiety was the most prevalent (93.2%) theme among middle-aged participants. Anxiety in the context of cyberbullying was characterized by a constant sense of worry and fear, both online and offline. Participants often described feeling on edge, unable to escape the tension caused by the possibility of encountering harmful comments or judgment, which affected their overall mental well-being.

Tiana expressed her growing sense of unease: “Every time I go online, I feel like something bad is going to happen. I’m constantly worried about the comments, the people who might say something hurtful. It started affecting me outside of the internet too. My heart races, I can’t focus on anything, and I’m always thinking about how to avoid confrontation. I can’t shake the feeling that something’s always wrong” (Tiana, female, 53 years old).

Jim shared how the constant pressure impacted his daily life: “Every time I see a notification, I feel this tightness in my chest. It’s like I can’t breathe sometimes. I used to feel comfortable online, but now, every post or comment feels like it could trigger something negative. I get anxious thinking about going out because I’m worried people will treat me differently, like they’ve already formed an opinion of me based on what they’ve seen online. It’s exhausting” (Jim, male, 46 years old). Cyberbullying deeply affects the mental well-being of individuals, manifesting as heightened anxiety that extends beyond the digital world into everyday life.

### 3.14. Theme 2 [Age 40–59]: Low Self-Esteem

Low self-esteem was the second most common (76.2%) theme among middle-aged participants. This theme was marked by a significant decrease in self-worth, marked by eroded confidence and seeing value in themselves. Participants often reported a persistent feeling of inadequacy and the inability to feel positive about themselves due to the harmful effects of cyberbullying.

Caroline explained how cyberbullying altered her perception of herself: “I used to feel proud of my achievements, even the small ones, but now, after all the hateful comments online, I feel like I’m not good enough for anything. I can’t shake the feeling that no matter what I do, someone will always have something bad to say. It’s like every little thing I do is under scrutiny, and I just don’t feel like I’m worth anything anymore” (Caroline, female, 50 years old).

Luke also discussed how cyberbullying had an impact on his self-worth: “It’s hard to look at myself in the mirror these days. I never used to think twice about how I looked, but now, after the constant mockery online, I feel like I’m always being judged. It doesn’t help that people don’t hold back their opinions about me—comments about my appearance and my age make me feel invisible, like I don’t matter at all” (Luís, male, 61 years old). Cyberbullying can leave individuals feeling insignificant, questioning their worth, and struggling with their sense of identity.

### 3.15. Theme 3 [Age 40–59]: Use of Substances

Very nearly three-quarters of participants mentioned the use of substances to cope with cyberbullying. Many reported turning to alcohol and tobacco to manage emotional pain. Individuals in this age group often resorted to substances to numb their feelings of anxiety, depression, and helplessness.

Paul spoke about how his increased use of alcohol helped him escape the constant negativity online: “I didn’t even realize it at first, but every time I saw one of those hateful comments or got one of those nasty messages, I’d reach for a drink. It started as just a way to forget, but now it feels like I can’t get through the day without it. I’ve tried cutting back, but the stress of dealing with it just makes me turn to the bottle again” (Paul, male, 58 years old).

Susan shared how she began smoking more frequently as a response to the bullying: “I never smoked much before, but after all the cruel things people said about me online, I started smoking more often. It felt like a quick way to calm down when I was feeling overwhelmed. And now, it’s hard to stop, and I know it’s not helping. I’ve got health problems already, but the bullying just pushed me further into this spiral” (Susan, female, 55 years old). Cyberbullying can push individuals toward unhealthy coping mechanisms. For these participants, the overwhelming stress of cyberbullying led them to rely on substances to self-manage their emotional pain, and as a less than desirable means of escape.

Finally, for participants aged 60 and older, the themes included: (a) frustration (78.1.%); (b) isolation (77.8%); and (c) disturbances in sleep and eating patterns (67.4%).

### 3.16. Theme 1 [Age 60+]: Frustration

The first theme, frustration, was mentioned by 78.1% of participants who were aged 60 and older. They expressed intense frustration due to their inability to effectively cope with or retaliate against cyberbullies. This frustration stemmed from their feeling of powerlessness in navigating the digital world, which only intensified the emotional toll of the online harassment they experienced.

Anthon shared how cyberbullying led him to feel trapped and frustrated: “I feel like I’m constantly battling to keep my peace of mind, but it’s like the moment I step online, everything goes wrong. I’ve tried to report it, but it never works. I can’t even figure out how to block people properly. I never imagined my retirement would be like this—so full of stress and frustration. I thought I could enjoy my retirement in peace, but now I feel angrier than ever” (Anthon, male, 70 years old).

Mara expressed the deep frustration she felt as a result of being targeted by cyberbullying: “I used to be proud of who I was and the things I could still do, but now I feel like I’m constantly being reminded of my age and how I don’t belong in the online world. At first, I tried to engage, but all I got were rude comments and insults. I never thought getting older would be so isolating, so full of this kind of frustration. I thought I could enjoy my time, but instead, it’s just constant negativity” (Mara, female, 67 years old). These quotes reflect the deep frustration felt by older participants who struggle to defend themselves in the face of cyberbullying. Their frustration was compounded by their limited digital literacy and lack of support in addressing the abuse, leaving them feeling helpless and overwhelmed.

### 3.17. Theme 2 [Age 60+]: Isolation

Feeling isolated was the second most prevalent (77.8%) theme among persons aged 60 and older. Many highlighted how cyberbullying impacted their mental health and led them to withdraw from their social circles. Cyberbullying increased their sense of isolation.

Brian explained how the bullying affected his sense of connection with others: “I used to enjoy chatting with old friends online, but now every time I go on, I just see those cruel messages. It feels like no matter where I go, I’m alone in this. I don’t even want to talk to my family about it. I feel like they wouldn’t understand what it’s like” (Brian, male, 70 years old).

Lily expressed how the cyberbullying made her withdraw even further: “I used to be very active in my church community and had a lot of friends I’d meet up with. But after the bullying started, I just couldn’t face them anymore. I’d stay home instead, and the isolation felt worse than the bullying itself. Now, I don’t talk to anyone as much; it’s like I’m fading away” (Lily, female, 77 years old).

Older people in this study were cutting themselves off from family, friends, and other communities. They experienced emotional harm and withdrew from social activities, with this leaving them lonely and without the everyday support that they had rightly relied on.

### 3.18. Theme 3 [Age 60+]: Disturbances in Sleep and Eating Patterns

The third theme, sleep and eating disorders, was mentioned by 67.4% of older participants. Cyberbullying significantly impacted their sleep patterns and eating habits. Some struggled to fall asleep or experienced a disturbed rest. Others mentioned turning to food for comfort and perhaps even eating more than usual. Others lost their appetite.

Filip shared how his sleep was affected: “I used to sleep well, but now it is difficult to have a full night of rest. I wake up every few hours, my mind racing with thoughts about what people said to me online. So, I wake up and go to check in the middle of the night. It’s like I can’t escape it, even in my sleep” (Filip, male, 72 years old).

Ariana talked about how the stress from bullying influenced her eating habits: “I’ve never been one to overeat, but for a while, I found myself eating just to fill the emptiness. It’s like food was the only thing that made me feel better, even if just for a few minutes. I’ve gained weight, and I don’t even enjoy it anymore. It’s just a way to distract myself from everything” (Ariana, female, 68 years old).

The stress and emotional pain experienced from cyberbullying is pervasive. It affected older participants’ ability to maintain everyday routines for maintaining some semblance of mental and physical health. For some, disrupted sleep and an unhealthy relationship with food were the new normal.

## 4. Discussion

This study sheds light on how cyberbullying rears its ugly head across different life stages, highlighting how these experiences are shaped by life stage. The three age groups that we studied emphasized unique challenges.

Participants between 18 and 39 years of age highlighted the emotional and social toll of cyberbullying. Their feelings of shame or humiliation highlight the profound impact of cyberbullying on a person’s self-image and self-worth. Social media is an extension of identity for many young adults (Stokes & Price, 2017), so when subjected to public mockery, it feels like a personal attack that reaches far beyond the individual (Al-Turif & Al-Sanad, 2023; Nixon, 2014). The results also suggest that these online experiences, particularly comments like those describing one’s appearance or personal views, can haunt individuals for weeks and feel even more serious because they are permanent, contributing to a negative self-concept (Nixon, 2014). Cyberbullying affords a larger audience and has staying power (Peebles, 2014). Additionally, cultural attitudes toward shame and dignity can also shape how individuals perceive and react to these experiences, potentially influencing their emotional responses to cyberbullying (Milosevic et al., 2023), highlighting the need for context-specific interventions in different demographic groups. Our participants felt as if they were earmarked and as moving targets.

The second theme, withdrawing from friends and family, suggests that cyberbullying can lead to significant real-world and online social isolation. Research shows that repeated harassment often drives individuals to self-isolate. Participants distanced themselves from their social circles to manage their emotional distress. Studies indicate that youth and younger adults perceive cyberbullying as having more severe repercussions than traditional bullying due to anonymity and broader reach (Macaulay et al., 2022). Often familiar social environments can be perceived as unsafe, prompting retreat (Coelho & Romão, 2018). Lack of support from friends and family can make young individuals more vulnerable to cyberbullying’s psychological effects, especially when older generations fail to see the internet as integral to daily life (Kahi et al., 2024).

Viewing harassment as positive and struggling with rules highlights how some young individuals perceive aggression and rule-breaking as ways to regain control in hostile virtual social environments. Harassment is often seen as acceptable and normalized online and not respecting rules becomes justified. Such behavior could be interpreted as an attempt to counteract feelings of powerlessness that often accompany bullying experiences (Higgins et al., 2020; Schwartz et al., 2001). Exposure to cyberbullying can make young people less persistent, more impulsive, and prone to anger and frustration (Favini et al., 2023). The emotional toll of bullying may drive them to exhibit aggressive behaviors to cope (Özer & Escartín, 2023). Diminished trust in authority figures, like parents or teachers, often worsens such mindsets, especially when steps taken to rectify cyberbullying are ineffective; rules and norms can seem irrelevant (Higgins et al., 2020).

In the 40–59 age group, the experience appears to be characterized by a loss of interest in hobbies, questioning about things they did or did not do and experiencing a sense of missing out.

Losing interest in hobbies emerged as a key theme among participants in the 40–59 age group, indicating that cyberbullying impacts social interactions and the pursuit of personal passions like hobbies. For this age group, hobbies can be a means of self-expression and a sense of connection with pleasurable activities (Iso-Ahola & Baumeister, 2023). When once pleasurable activities become a vehicle for criticism, individuals may withdraw, either to avoid further bullying or due to a reduced sense of enjoyment (Wolke & Lereya, 2015). Disrupted routines can erode midlife adults’ self-esteem. Additionally, demographic characteristics such as socio-economic status may influence these experiences, as individuals in different socio-economic groups might have varying access to resources and social support that could buffer the impact of cyberbullying (Hellfeldt et al., 2019).

The next theme, questioning about things you did or did not do, highlights the complex emotional toll of cyberbullying, with a stronger focus on questioning one’s choices, and feelings of regret or self-blame. Middle-aged adults often place significant value on maintaining their reputation and relationships, both online and offline (Jiang & Song, 2022). When online interactions go awry, they may internalize criticism and question their actions or inactions. This guilt can lead to a cycle of avoidance, where individuals withdraw from online spaces entirely to escape further scrutiny or shame. However, such withdrawal often exacerbates feelings of regret, as they may feel they missed opportunities to repair relationships or to stand up for themselves (Firth et al., 2024).

The theme of the feeling of missing out highlights the human need for belonging. A sense of belonging is vital for psychological growth and for feeling valued (Beyens et al., 2016; Shahzadi et al., 2024). Participants expressed that being excluded from known others’ social interactions online while being excluded left them feeling isolated and undervalued. This aligns with Przybylski et al.’s (2013) definition, which links the feeling of missing out to anxiety and stress from the fear of missing rewarding experiences. Online platforms, meant for connection, can paradoxically become distressing spaces, amplifying fears of social exclusion and a lack of significance (Casale & Flett, 2020; Gioia et al., 2021a). Interventions focused on improving social support networks could alleviate these feelings, offering opportunities for individuals to engage in both online and offline spaces where they can feel included and valued. These interventions could involve fostering digital literacy, self-worth enhancement, and peer-led groups for more inclusive virtual communities.

For individuals aged 60 and older, cyberbullying is marked by negative thoughts and self-talk, a heightened sensitivity to judgment, and financial vulnerability.

Most notable to older participants was negative thinking and self-talk, highlighting the internal struggles many face when targeted by cyberbullies. Participants described recurring self-criticism and internalized negativity, worsened by harmful comments or exclusion online. Older adults are particularly vulnerable to negative self-perceptions from social rejection or criticism (Dias & Fraga, 2024). Cyberbullying intensifies feelings of inadequacy and self-doubt, as negative interactions may confirm existing insecurities about aging, technology use, or social relevance. This internalization erodes older people’s confidence in online environments and impacts their self-worth (Hu & Li, 2022; Kim et al., 2021).

The second theme, feeling judged negatively, highlighted concerns about how participants were perceived in digital spaces. Many felt scrutinized or belittled, often interpreting online interactions as critical or dismissive. This aligns with research suggesting older adults may be more sensitive to social evaluation, especially in environments where they feel less competent, like digital platforms (von Humboldt et al., 2022b). Cyberbullying reinforced the perception of being unwelcome or incapable in digital conversations. This may stem from societal narratives marginalizing older adults as less tech-savvy, making even small criticisms feel personal and upsetting (NORC, 2021; von Humboldt et al., 2022b). Such responses limit their ability to connect with others and hinder digital inclusion.

The third theme among the older age group was feeling financially vulnerable. Participants expressed concerns about protecting themselves from online scams, which disproportionately target older adults due to limited digital literacy and greater trust in perceived authorities (NORC, 2021). Financial vulnerability affects their sense of security and life satisfaction. Research suggests that perceived financial well-being is more important for life satisfaction than actual financial resources (Yeo & Lee, 2019), indicating that the psychological toll of financial insecurity, amplified by cyberbullying, can undermine older adults’ well-being, even if their financial resources are sufficient.

The mental health consequences of cyberbullying varied across age groups. In the 18–39 age group, depressive symptoms, being easily angered, and suicidal behavior were prominent. Cyberbullying has profound psychological impacts during this life stage.

Depressive symptoms took shape as feelings of sadness, low energy, and worthlessness, highlighting the emotional toll of negative online interactions. Cyberbullying often targets self-esteem, worsening feelings of inadequacy and social rejection (Kowalski et al., 2018). The constant connectivity of digital platforms makes bullying feel inescapable, deepening helplessness and contributing to depression (Kowalski et al., 2018; Selkie et al., 2015). A positive correlation was also found between daily social media use and depression (Yoon et al., 2019). Unlike face-to-face bullying, online harassment persists, making it harder for younger adults to escape without withdrawing socially.

Easy anger emerged as the second theme, where participants described feelings of heightened irritability and frustration, often lashing out at others or internalizing their anger. Research suggests that persistent exposure to online hostility can amplify stress responses, leading to emotional dysregulation (Gioia et al., 2021b). For younger adults, who may not yet have fully developed coping mechanisms, cyberbullying can create a cycle of negative emotions. The anonymity of online interactions often worsens this issue, as it strips away social accountability and intensifies the perceived aggression. This lack of face-to-face interaction can make online hostility feel more direct and personal, triggering disproportionate emotional reactions such as anger (Alia-Klein et al., 2020; Gioia et al., 2021b). Additionally, anger can be both a coping mechanism and a tension release. For some, expressing anger is an attempt to regain control in a situation where they feel powerless (O’Grady et al., 2012).

The third theme, suicidal behavior, was a deeply troubling finding among younger adults who had experienced cyberbullying. Participants recounted feeling overwhelmed by despair, with some expressing thoughts of self-harm or attempting suicide. Additionally, the challenges on social media—such as the normalization of harmful behaviors—were also mentioned, where individuals may not fully understand the seriousness of cyberbullying, engaging in it simply because others are doing so. The literature shows a strong association between cyberbullying and suicidal ideation, as well as links with factors such as feelings of hopelessness, social isolation, and diminished self-worth (Fekih-Romdhane et al., 2024; Nixon, 2014). Unlike traditional bullying, cyberbullying can invade private spaces, making victims feel relentlessly exposed and vulnerable, pushing them toward a mental health crisis (Bansal et al., 2023; Nixon, 2014). Interventions for this age group could focus on improving emotional regulation skills, and providing mental health support to address the emotional and psychological effects of cyberbullying.

Among the 40–59 age group, anxiety, diminished self-esteem, and increased use of substances suggest a struggle to cope with bullying through self-medication and a decline in self-worth.

For participants between 40 and 59 years of age, anxiety was the most prevalent theme. Participants described persistent unease, stress, and worry about their online interactions, which affected other areas of their lives. This aligns with research showing that cyberbullying can trigger significant anxiety, especially for those already facing stress from work, family, or health concerns (National Institute of Mental Health, 2022; von Humboldt et al., 2019). For middle-aged adults, digital interactions might serve as a space for connection or professional engagement (Chase et al., 2022) and cyberbullying disrupts these, creating insecurity. Unlike younger individuals, middle-aged adults may view cyberbullying as a threat to their social or professional stability and identity, increasing stress and negatively impacting their well-being (Snyman & Loh, 2015). Interventions could focus on stress-management strategies, such as mindfulness or therapy, to help middle-aged adults manage anxiety triggered by digital interactions.

The next theme identified was low self-esteem, highlighting how cyberbullying can erode individuals’ sense of self-worth. Midlife adults face age-related changes, career pressures, and familial and societal expectations (Wagner et al., 2014). Middle-aged adults often derive self-esteem from their roles and responsibilities, such as professional accomplishments or family leadership (Wagner et al., 2014). Cyberbullying can target these very aspects, amplifying feelings of inadequacy and failure. For example, being ridiculed online for their appearance, opinions, or perceived outdatedness might leave individuals questioning their value or relevance in personal and professional spheres (Peebles, 2014). Research strongly links feeling excluded from things due to age or from friendships with self-deprecation (Low et al., 2023; von Humboldt et al., 2023a, 2023b).

The third theme was the use of substances, with participants revealing that tobacco and alcohol were often used as mechanisms to cope with the stress and emotional turmoil caused by cyberbullying. This behavior aligns with broader research indicating that individuals under significant stress often resort to substance use to manage their emotional pain or to escape from overwhelming situations (American Addiction Centers, 2024). For middle-aged adults, for instance, being publicly ridiculed or attacked online can lead to feelings of shame or embarrassment that they may not feel comfortable discussing, prompting them to seek solace in tobacco or alcohol (Ostroff et al., 2022). However, substance use can worsen the long-term effects of bullying, damaging physical health and impairing judgment, potentially complicating personal and professional situations (Brevers et al., 2014).

For participants aged 60 and older, frustration, feelings of isolation, and disruptions in sleep and eating patterns highlight the compounded vulnerabilities faced by older individuals in navigating online harassment.

First and foremost, participants aged 60 and older expressed frustration. Frustration in this context stemmed from several sources, including technological challenges, difficulty in understanding the intentions behind harmful comments, and feelings of being misunderstood or undervalued. Unlike younger individuals who might adapt quickly to the evolving norms of digital communication, older adults often find themselves excluded or targeted due to their perceived lack of digital fluency (Tomczyk et al., 2023). Digital services evoke a wide range of emotions for older adults—frustration, desperation, confusion, and even fear. However, these experiences can also lead to feelings of competence, pleasantness, and pride when positive outcomes are achieved (Valkonen & Kujala, 2024; von Humboldt et al., 2022a). However, when cyberbullying incidents ridicule their mistakes or dismissively group all older people into the same stereotype, they amplify frustration. Such attacks strike at their sense of autonomy and competence, undermining the confidence they may have built through digital engagement.

The second theme identified was, a feeling expressed by a substantial portion of respondents. Online bullying exacerbates existing feelings of loneliness and exclusion, especially for older adults who already face reduced social interactions due to life circumstances like retirement, loss of loved ones, or physical mobility limitations. The transition to digital communication as a primary means of social connection can be particularly challenging for this age group (Ang, 2022). Cyberbullying incidents further alienate older individuals, leading them to withdraw from digital spaces entirely. This withdrawal compounds their sense of isolation, as they lose a crucial avenue for maintaining relationships and participating in community life. Social isolation for older people is not merely a result of physical separation but often stems from a perceived lack of meaningful connections (Ang, 2022; Sen et al., 2022; von Humboldt et al., 2018).

With respect to our final theme, older people’s concerns centered around sleep and food. They had difficulties falling asleep or maintaining regular sleep patterns due to ruminating thoughts about online interactions. For some, the fear of encountering further negative experiences online led to heightened stress levels, which affected their ability to relax (Evangelou et al., 2024). Changes in eating habits, such as loss of appetite or emotional eating, were coping mechanisms tied to feelings of distress or inadequacy (Arjmand et al., 2023). Research indicates that nighttime phone use may exacerbate the effects of cyberbullying on sleep and psychological well-being (Centofanti et al., 2024). Additionally, cyber victimization has been linked to eating disorders psychopathology, highlighting its broad impact on mental and physical health (Marco & Tormo-Irun, 2018). Tailored interventions should focus on improving digital literacy and providing clear guidance on navigating online spaces. Psychoeducation programs that help older adults understand the impact of cyberbullying and promote emotional resilience could also be crucial in mitigating feelings of frustration (Marco & Tormo-Irun, 2018).

This study casts light on the various important impacts of cyberbullying across three different generations; however, it has several limitations. A reliance on self-reported data introduces the possibility of response bias, as participants may have underreported or exaggerated their experiences due to social desirability or recall issues. For example, midlife participants spoke of lingering shame and regret. Additionally, while qualitative design allowed for an in-depth exploration of participants’ perceptions and experiences, it limits the generalizability of the findings to a broader population. Second, this is a cross-sectional study. Causal inferences about cyberbullying in relation to, for example, anxiety, low self-esteem, and substance use require longitudinal research. How these effects evolve over time and whether they differ by age group or other demographic variables warrants further empirical attention. Moreover, this study does not account for the role that cultural, economic, or technological factors could play in shaping cyberbullying experiences. Differences in internet access, social media usage patterns, or cultural attitudes toward bullying might shape how participants perceive and respond to these incidents. Additionally, the study may have overlooked important protective factors, such as resilience, family support, or coping mechanisms, that could mitigate the impact of cyberbullying. Future research should incorporate these elements to provide a more comprehensive understanding of the phenomenon and inform interventions designed to support affected individuals. An in-depth analysis of societal influences, such as cultural norms, ageism, and social media trends, would further illuminate how these external factors shape the experiences and perceptions of cyberbullying, deepening our understanding of the reported issues. Furthermore, a comparative cross-cultural perspective could help clarify how socio-cultural dynamics influence cyberbullying experiences and coping strategies across different global regions. Longitudinal studies tracking the long-term mental health effects of early cyberbullying experiences would also offer valuable insights. By integrating psychological resilience factors with digital literacy interventions, future research can contribute to a more effective framework for addressing the relationship between online harm and mental health across different life stages.

Notwithstanding these limitations, the findings of this study have important implications for both research and practice. The differences in how cyberbullying affects various age groups highlight the need for age-specific interventions. Younger individuals may benefit from resilience-building and social support strategies, while middle-aged adults might need interventions addressing workplace stress and social standing. For older adults, enhancing digital literacy and protective measures against online exploitation could be key.

These results emphasize the importance of addressing cyberbullying as a multifaceted issue beyond adolescence. Employers, educators, and policymakers should include provisions for cyberbullying in anti-bullying policies to protect employees’ psychological well-being. Mental health professionals must consider the unique stressors of online interactions in their therapeutic approaches.

This study also underscores the role of social media platforms in both perpetuating and mitigating cyberbullying. Platforms should strengthen measures to detect and prevent bullying, offering accessible reporting tools and mental health resources. Educational campaigns promoting digital etiquette and empathy could help reduce cyberbullying across all age groups. Social platforms are meant for social connectedness, not social disregard. For some, virtual spaces may be the only social spaces for connecting with others.

## 5. Conclusions

Cyberbullying infiltrates the lives of younger, middle-aged, and older individuals in uniquely harmful ways. This study sheds light on the far-reaching impacts of cyberbullying, including disrupted social connections, undermined self-worth, and exacerbated vulnerability to physical, financial, and mental harms across different stages of life. Cyberbullying is not merely a modern adaptation of traditional bullying, but a distinct phenomenon shaped by the digital relational dynamics, requiring tailored strategies for prevention and intervention.

In sum, several key themes emerged across age groups, illustrating the nuanced effects of cyberbullying. Younger individuals spoke of feeling ashamed or humiliated, withdrawing from friends and family, and of viewing harassment as positive while struggling with rules. They also reported depressive symptoms, easy anger, and suicidal behavior, emphasizing the emotional toll cyberbullying takes on their well-being and relationships. A loss of interest in beloved leisure pursuits, questioning one’s actions or inactions, and a sense of missing out mattered to midlife adults. Felt anxiety, low self-esteem, and substance use reflect how cyberbullying disrupts social interactions and amplifies stress, especially in professional contexts. Older adults reported negative thoughts and self-talk, feeling judged negatively, and feeling financially vulnerable. Frustration, isolation, and sleep and eating disorders further illustrate the intersection of cyberbullying with age-related challenges.

In the digital age, where online interactions are part-and-parcel of the social fabric of people’s everyday lives and livelihoods, understanding and addressing cyberbullying is paramount. This study highlights that it is important to not only mitigate harm but to also create supportive digital spaces where individuals of all ages can connect, grow, and thrive.

## Figures and Tables

**Table 1:** Sample of sociodemographic and health characteristics.

Characteristics	Younger Adults 301 (34.1)		Middle Adults 294 (33.3)		Older Adults 288 (32.6)		Total 883 (100.0)	
Age (Average ± *SD*)	46.4 ± 6.841							
Gender, *n* (%)								
Female	157	(52.2)	152	(51.7)	149	(51.7)	458	(51.9)
Male	144	(47.8)	142	(48.3)	139	(48.3)	425	(48.1)
Education, *n* (%)								
Primary school	42	(14.0)	31	(10.5)	91	(31.6)	164	(18.6)
Middle school	144	(47.8)	134	(45.6)	112	(38.9)	390	(44.2)
≥High school	115	(38.2)	129	(43.9)	85	(29.5)	329	(37.3)
Marital Status, *n* (%)								
Married or in a relationship	147	(48.8)	174	(59.2)	187	(64.9)	508	(57.5)
Not married nor in a relationship	154	(51.2)	120	(40.8)	101	(35.1)	375	(42.5)
Living arrangements, *n* (%)								
Live with someone	179	(59.5)	237	(80,6)	194	(67.4)	610	(69.1)
Live alone	122	(40.5)	57	(19.4)	94	(32.6)	273	(30.9)
Family Annual Income, *n* (%)								
≤25,000 €	155	(51.5)	181	(61.6)	157	(54.5)	493	(55.8)
>25,000 €	146	(48.5)	113	(38.4)	131	(45.5)	390	(44.2)
Perceived health, *n* (%)								
Good	247	(82.1)	201	(68.4)	174	(60.4)	622	(70.4)
Poor	54	(17.9)	93	(31.6)	114	(39.6)	261	(29.6)

**Table 2:** Overview of themes identified.

Objective 1: How cyberbullying is emotionally experienced	
18–39 age group	Feeling ashamed or humiliated (92.4%)
	Withdrawing from friends and family (81.1%)
	Experiencing harassment as positive and difficulties with rules (80.7%)
40–59 age group	Losing interest in hobbies (89.5%)
	Questioning about things they did or did not do (76.9%)
	Experiencing a sense of missing out (74.8%)
60+ age group	Negative thoughts and self-talk (91.3%)
	Feeling judged negatively (87.5%)
	Feeling financially vulnerable (86.1%)
Objective 2: Impact of cyberbullying on mental health	
18–39 age group	Depressive symptoms (79.7%)
	Easy anger (76.1%)
	Suicidal behavior (43.2%)
40–59 age group	Anxiety (93.2%)
	Low self-esteem (76.2%)
	Use of substances (74.8%)
60+ age group	Frustration (78.1.%)
	Isolation (77.8%)
	Disturbances in sleep and eating patterns (67.4%)

## Data Availability

Data transparency: the data that support the findings of this study are available from the corresponding author, [SvH], upon reasonable request. Software application or custom code: No software application or custom code was used for the coding process.
